# The prognostic role of hemoglobin levels in patients undergoing concurrent chemo-radiation for anal cancer

**DOI:** 10.1186/s13014-018-1035-9

**Published:** 2018-05-02

**Authors:** Pierfrancesco Franco, Francesco Montagnani, Francesca Arcadipane, Chiara Casadei, Kalliopi Andrikou, Stefania Martini, Giuseppe Carlo Iorio, Mario Scartozzi, Massimiliano Mistrangelo, Lorenzo Fornaro, Paola Cassoni, Stefano Cascinu, Umberto Ricardi, Andrea Casadei Gardini

**Affiliations:** 1 Department of Oncology, Radiation Oncology, University of Turin at AOU Citta’ della Salute e della Scienza, Via Genova 3, 10126 Turin, Italy; 2 Department of Oncology, Medical Oncology, ASL Biella, Biella, Italy; 3 Department of Oncology, Radiation Oncology, AOU Citta’ della Salute e della Scienza, Turin, Italy; 4 Department of Medical Oncology, Istituto Scientifico Romagnolo per lo Studio e la Cura dei Tumori (IRST) IRCCS, Meldola, Italy; 5 Modena Cancer Center, Department of Oncology/Hematology, University of Modena and Reggio Emilia, Modena, Italy; 6 Department of Medical Oncology, University of Cagliari, Cagliari, Italy; 7 Department of Surgical Sciences, University of Turin, Turin, Italy; 8 Unit of Medical Oncology 2, Azienda Ospedaliero-Universitaria Pisana, Pisa, Italy; 9 Department of Medical Sciences, Pathology Unit, University of Turin, Turin, Italy

**Keywords:** Anal cancer, Hematologic toxicity, Anemia, Hemoglobin, Prognostic factors

## Abstract

**Background:**

Concurrent chemo-radiation (CT-RT) is a standard therapy for squamous cell carcinoma of anal canal. Different clinical and biological factors may potentially affect outcome. We investigated the prognostic role of baseline hemoglobin (Hb) in a cohort of anal cancer patients submitted to CT-RT with 5-fluorouracil and mitomycin C.

**Methods:**

Up to 161 patients with clinical stage T1-T4/N0-N3/M0 were treated. Response was assessed at 6 weeks and thereafter at 3, 6 and 12 months. Two different approaches were used:a)simultaneous integrated boost following RTOG 05-29 indications;b)first sequence of 45Gy/25 fractions to the pelvis followed by 9–14.4 Gy/5–8 fractions to the macroscopic disease. Primary endpoints were progression-free survival (PFS) and overall survival (OS).

**Results:**

On multivariate analysis, pre-treatment Hb level had a significant correlation to OS (HR:0.53;95% CI:0.33–0.87; *p* = 0.001), but not to PFS (HR:0.78;95% CI:0.53–1.15; *p* = 0.12) Patients with pre-treatment Hb ≥ 12 g/dl had 5-year PFS and OS of 82.2%, compared to 29.3% and 32.8% for those below the threshold. The likelihood to achieve a complete remission increased by 5.6% for every single-unit (g/dl) increase in baseline Hb level over 11 g/dl. On multivariate analysis, response to treatment had a significant correlation to PFS (incomplete vs complete response – HR:5.43;95% CI:2.75–10.7; *p* < 0.0001) and OS (HR: 6.96;95% CI:2.96–16.5; *p* < 0.0001).

**Conclusions:**

We showed that baseline Hb level is a strong indicator for poor response to RT-CT in anal cancer patients. A close clinical monitoring for incomplete response to treatment should be advised in patients with low pre-treatment Hb. The hypothesis that the preservation of adequate Hb level during treatment may lead to a better outcome needs prospective evaluation.

## Background

Anal cancer is considered a rare tumor, accounting for 6% of all malignancies arising in the ano-rectal region [1]. Concurrent chemo-radiation (CT-RT) is the standard therapeutic option in this setting, providing consistent clinical outcomes [2, 3]. Prospective trials reported loco-regional recurrence (LRR) rates ranging from 60% to 80% and overall survival (OS) rates from 65% to 78% at 3–5 years, depending on disease and patient characteristics [4–8]. Given the relatively infrequent occurrence of squamous cell carcinoma of the anal canal, the correlation between clinical factors and outcome has always been challenging to assess [9]. Most of the factors explored are related to tumor such as size and nodal status, while patient features are mostly related to gender and race [10]. Some of the randomized phase III trials who set the standard for the treatment of anal cancer, such as the European Organization for Research and Treatment of Cancer (EORTC) trial 22861, the Radiotherapy and Oncology Group (RTOG) trial 98-11 and the Anal Cancer Trial-I study (ACT-I) provided analyses on prognostic factors [5, 7, 11]. The EORTC 22861 has shown that male sex, nodal involvement and skin ulceration are independent predictors of LRR and OS [5]. The RTOG 98–11 trial found out a significant correlation between male sex and nodal involvement and LRR and established tumor size (> 5 cm) as an independent predictor of disease-free-survival (DFS) and OS [12]. Mature outcomes from the ACT-I trial supported evidence for palpable lymphnodes and male sex as prognostic factors for LRR and OS and interestingly, showed that lower baseline hemoglobin levels could predict for the risk of cancer-related death and death from any cause [11]. It is a common clinical observation that anemia is frequent in cancer patients [13]. This could substantially increase the proportion of hypoxic cancer cells, leading to an intrinsic resistance to radiotherapy (RT) and chemotherapy (CT), with potential detrimental effects on treatment outcomes [14]. The impact of hemoglobin level in anal cancer patients submitted to CT-RT has been rarely reported [13, 15]. In the present study, we intended to investigate the influence of different clinical prognostic factors, particularly baseline hemoglobin (Hb) levels, on treatment outcomes within a cohort of anal cancer patients submitted to combined RT and CT.

## Methods

### Patient selection

We retrieved clinical data of patients treated for anal cancer at the Radiation and Medical Oncology Departments of 3 Italian institutions, namely University of Turin, AOU Citta’ della Salute e della Scienza in Turin, Istituto Scientifico Romagnolo per lo Studio e la Cura dei Tumori (IRST) in Meldola and Modena Cancer Center in Modena. Briefly, all patients had a histologically confirmed diagnosis of squamous cell carcinoma located either within the anal canal or margin. Tumor stage was defined following the indications of the American Joint Committee on Cancer (2002 version) and patients with clinical stage T1-T4, N0-N3, M0 were included. Patients having clinical T1 N0 tumors of the anal margin were excluded, because generally treated with local excision.

### Staging

Pre-treatment clinical evaluation included complete medical history, physical examination and complete laboratory testing. Staging included a chest, abdomen and pelvis computed tomography (CT) scan and a magnetic resonance imaging (MRI) of the pelvis with the adjunct of positron-emission tomography (PET) and/or inguinal sentinel lymphnode biopsy (SLNB) for systemic and nodal staging in specific cases.

### Patient evaluation

Patients were followed-up according to local practice and vital status was clinically updated in 2018. Response to treatment was assessed at 4 time-points, namely at 6 weeks after CT-RT and thereafter at 3, 6 and 12 months [16]. Assessment comprised clinical and digital rectal examination, pelvic MRI and CT scan of the upper abdomen and thorax (for staging completion). Biopsy of any suspicious lesion detected at endoscopic examination was undertaken. Patients achieving a complete response (CR) were classified as ‘complete responders’ in case of clearance of all macroscopic disease for both primary tumor and regional lymphnodes and negative biopsy examination (when biopsy was performed). In case of residual tumor on imaging or positive biopsy after 6 months from CT-RT completion, patients having less than complete response (IR), were classified as ‘incomplete responders’. Written informed consent for treatment was obtained for all patients. The Ethical Review Board of each Institutional Hospital approved the present study.

### Radiotherapy

Two different approaches were used. At the University of Turin, patients were submitted to a simultaneous integrated boost (SIB)- based RT strategy and dose prescription was set according to the RTOG 05-29 indications modulated on clinical stage at presentation [17]. Patients with cT2N0 disease were given 50.4 Gy in 28 fractions (1.8 Gy daily) to the primary anal tumor, while the elective nodal volume was prescribed 42 Gy in 28 fractions (1.5 Gy/daily). Patients presenting cT3-T4/N0-N3 disease were prescribed 54 Gy in 30 fractions (1.8 Gy daily) to the gross tumor volume, while gross nodal disease was prescribed 50.4 Gy in 30 fractions (1.68 Gy daily) if sized ≤ 3 cm or 54 Gy in 30 fractions (1.8 Gy daily) if > 3 cm. Elective nodal volume was prescribed 45 Gy in 30 fractions (1.5 Gy daily) [14, 15]. Details and results of this treatment strategy have been previously published [18–20]. Patients treated at IRST in Meldola and Modena Cancer Center in Modena were given a first RT sequence of 45 Gy in 25 fractions (1.8 Gy daily) delivered over 5 weeks to the macroscopic primary and nodal tumor and prophylactic volumes (pelvic and inguinal nodes, ischio-anal fossa and mesorectum). In the second sequence, an adjunctive dose of 9–14.4 Gy in 5–8 fractions was delivered sequentially to the macroscopic disease up to a total nominal dose of 54–59.4 Gy.

### Chemotherapy

All patients were treated according to the Nigro regimen. Hence, concomitant CT consisted of 5- fluorouracil (5-FU) (1000 mg/m^2^/day) given as continuous infusion for 96 h (days 1–5 and 29–33) combined with mitomycin C (MMC) (10 mg/m^2^) given as bolus (days 1 and 29). Mitomycin C was capped at 20 mg maximum dose. A total of 2 concurrent cycles were planned for each patient.

### Statistical analysis

Baseline characteristics and clinical endpoints are presented for all patients. Discrete and continuous variables were summarized by frequencies and percentages and using standard measures of central tendency and dispersion (mean and standard deviation). The time-to-event functions were estimated by the Kaplan-Meier product-limit method. Cox proportional-hazard models were used to estimate the Hazard Ratios (HR) and the associated 95% confidence interval (95%CI) both for the univariate and multivariate analysis. We used different analytic strategies for multivariate model implementation, namely forward, backward and stepwise approaches. Wald test and likelihood ratio tests for the case of nested models were used to assess significance of both single covariates and the model as a whole. Proportional hazard assumption was tested with visual inspection of log-log survival curves, plotted scaled Schoenfen’s residuals and global Kolmogorov-Smirnov test. Collinearity among independent variables was evaluated with Fisher’s exact test, t-test of difference between means and ANOVA, depending on the nature of the covariate. The following variables were investigated: age, gender, tumor and nodal stage, response to treatment, and overall treatment duration. Primary endpoints were progression free survival (PFS) and OS. Progression free survival was defined by the time interval between diagnosis and disease recurrence and/or progression at any site, death or lost at follow-up. Conversely, OS was calculated from the date of diagnosis to that of death from any cause or lost at observation. To assess the eventual correlation between the chance to achieve a CR and baseline Hb values, weighted linear regression was performed with pre-treatment Hb values as independent variable and the CR rate in predefined group of patients as dependent variable. Patients were divided in 5 categories based on baseline Hb values (< 11 g/dl; 11–12 g/dl; 12–13 g/dl; 13–14 g/dl; > 14 G/dl). All the analyses were performed with ‘rms’ and ‘survival’ packages of R software environment (https://www.r-project.org).

## Results

A total of 161 patients was analysed from 3 centers (49, 96 and 16 patients, respectively). No significant difference was found in terms of patient characteristics among the Institutions. Specifically, mean Hb values at baseline were 13.20 g/dl (SD: ± 1.44), 12.90 g/dl (SD: ± 1.57) and 12.85 g/dl (SD: ± 2.20), respectively (*p* = 0.56).

Most of the patients were female (74.5%), HIV negative (94.4%) with a mean age of 62. Most represented single global tumor stage was stage II (44.7%), but locally advanced disease (stage IIIA and IIIB) was seen in up to 46.6% of patients (Table 1). Most of the patients (59.6%) were treated with a SIB approach, with up to 54 Gy to the macroscopic tumor disease (60.4%). Those treated with a sequential boost approach underwent a 9 Gy boost in 65.1% of the cases.

**Table 1:** Patient and tumor characteristics

Variable	N (%)
Age	
*Mean*	62
*Range*	36–83
Sex	
*Female*	120 (74.5)
*Male*	41 (25.5)
HIV status	
*Positive*	9 (5.6)
*Negative*	152 (94.4)
T-stage	
*T1*	14 (8.7)
*T2*	90 (56.0)
*T3*	40 (24.8)
*T4*	15 (9.3)
*NA*	2 (1.2)
N-stage	
*N0*	91 (56.6)
*N1*	26 (16.1)
*N2*	34 (21.1)
*N3*	10 (6.2)
Global stage	
*I*	13 (8.1)
*II*	72 (44.7)
*IIIA*	29 (18.0)
*IIIB*	46 (28.6)
*NA*	1 (0.6)
Grading	
*G1*	12 (7.5)
*G2*	86 (53.4)
*G3*	45 (27.9)
*NA*	18 (11.2)

*N* number, *T-stage* tumor stage, *N-stage* nodal stage, *NA* not available

Almost all patients (98.1%) were given 2 cycles of 5-FU and MMC. Mean baseline Hb was 13.1 g/dl, while at the end of treatment mean value was 11.6 g/dl (Table 2). With anemia as endpoint, acute hematologic toxicity was ≥ G2 in 10% of the patients (Table 2).

**Table 2:** Hemoglobin levels and grade of anemia

Hb values		
	Pre-treat	Post-treat
*Mean (g/dl)*	13.11	11.63
*Range (g/dl)*	7.63–16.22	8.44–14.71
Anemia (CTCAE v4.02) - N(%)		
NA	G0-G1	G2-G3
10 (6.2)	135 (83.8)	16 (10)

*Hb* hemoglobin, *g/dl* grams/deciliter, *pre-treat* pre-treatment, *post-treat* post-treatment, *N* number, *CTCAE v4.02* Common Toxicity Criteria for Adverse Effects version 4.02

Objective response, evaluated at the planned time-point after the end of CT-RT, highlighted CR in 76.4% of patients and IR in 23.6%, respectively (Table 3). After a median follow up of 27 months (range: 1–30), the 3-year PFS and OS were 71.9% (95% CI:64.2%–80.5%) and 83.1% (95% CI:76.4%–9.5%), respectively. Five-year PFS and OS were 71.9% (95% CI: 64.2%–80.5%) and 76.1% (95% CI: 67.3%–86.0%).

**Table 3:** Objective response rate

Objective response	
	*N(%)*
*CR*	123 (76.4)
*IR*	38 (23.6)

*CR* complete remission, *IR* incomplete response

On univariate analysis, considering Hb as a continuous variable, a higher baseline Hb level significantly affected PFS (HR:0.57;95% CI:0.39–0.85;*p* = 0.049) and OS (HR:0.53;95% CI:0.29–0.96; *p* = 0.047). Moreover, achieving a CR after CT-RT significantly affected outcomes (Table 2). Specifically, obtaining a CR was significantly associated to improved PFS (HR: 5.39;95% CI:2.79–10.40; *p* < 0.0001) and OS (HR: 6.26;95% CI:2.73–14.40; *p* < 0.0001) (Tables 4 and 5).

**Table 4:** Univariate and multivariate analysis for Overall Survival

	Univariate Analysis		Multivariate Analysis	
Variable	*HR (95% CI)*	*p-value*	*HR (95% CI)*	*p-value*
Age > 65	1.44 (0.64–2.43)	0.58	NA	NA
Male sex	2.23 (1.42–3.05)	0.01	3.66 (1.56–8.60)	0.002
G3 vs G1 G2 vs G1	1.36 (0.58–3.21) 0.33 (0.03–2.92)	0.29 0.32	NA	NA
T3-T4 vs T1-T2	1.94 (0.88–4.25)	0.12	NA	NA.
N + ve vs N -ve	2.11 (1.31–2.90)	0.02	2.25 (1.00–5.17)	0.049
RT total dose	1.42 (0.87–2.33)	0.16	NA	NA
Boost: yes vs no	1.72 (0.40–7.35)	0.47	NA	NA
OTT > 42 days	1.75 (0.86–2.65)	0.19	NA	NA
Hb	0.5 (0.30–0.83)	0.006	0.53 (0.33–0.87)	0.001
Response	6.26 (2.73–14.40)	< 0.0001	6.96 (2.96–16.50)	< 0.0001

*RT* Radiotherapy, *Hb* Basal haemoglobin levels (gr/dl), *Boost* Radiotherapy boost: given vs not, *Response* Incomplete response vs complete response, *OTT* Total legth of chemo-radiation treatment, *N* node, *+ve* positive, *−ve* negative, *HR* hazard ratio, *CI* confidence interval, *NA* not available

**Table 5:** Univariate and multivariate analysis for Progression Free Survival

	Univariate Analysis		Multivariate Analysis	
Variable	*HR (95% CI)*	*p-value*	*HR (95%CI)*	*p-value*
Age > 65	1.04 (0.39–1.70)	0.82	NA	NA
Male sex	1.17 (0.44–1.90)	0.52	NA	NA
G3 vs G1 G2 vs G1	1.87 (0.90–3.88) 1.76 (0.51–6.10)	0.92 0.38	NA NA	NA NA
T3-T4 vs T1-T2	1.73 (0.89–3.33)	0.11	NA	NA
N + ve vs N -ve	2.16 (1.18–3.96)	0.012	1.98 (1.01–3.88)	0.046
RT total dose	1.12 (0.79–1.58)	0.52	NA	NA
Boost: yes vs no	0.84 (0.32–2.18)	0.72	NA	NA
OTT > 42 days	1.18 (0.53–1.85)	0.38	NA	NA
Hb	0.57 (0.39–0.85)	0.005	0.78 (0.53–1.15)	0.12
Response	5.39 (2.79–10.4)	< 0.0001	5.43 (2.75–10.70)	< 0.0001

*RT* Radiotherapy, *Hb* Basal haemoglobin levels (gr/dl), *Boost* Radiotherapy boost: given vs not, *Response* Incomplete response vs complete response, *OTT* Total legth of chemo-radiation treatment, *N* node, *+ve* positive, *−ve* negative, *HR* hazard ratio, *CI* confidence interval, *NA* not available

Results of the multivariate analyses were almost identical despite the different methods used (forward, backward and stepwise). On multivariate analysis, pre-treatment Hb level had a significant correlation to OS (HR:0.53;95% CI:0.33–0.87;*p* = 0.001), but not to PFS (HR:0.78;95% CI:0.53–1.15;*p* = 0.12) (Tables 4 and 5). Response to treatment maintained a significant correlation to PFS (incomplete vs complete response – HR:5.43;95% CI:2.75–10.7; *p* < 0.0001) and OS (HR: 6.96;95% CI:2.96–16.5; *p* < 0.0001). Comparison of mean pre-treatment Hb values in patients having a CR (mean Hb: 13.2 g/dl; SD: ± 1.12) or IR (mean Hb: 12.2 g/dl; SD: ± 1.86) showed a significant difference (t-test; *p* value = 0.043). We also detected a significant correlation between basal Hb levels and lymph node status (*p* = 0.02). Both variables retained statistical significance in the multivariate model, suggesting an independent effect. In adjunct, the Variance Inflation Factor (VIF) was 1.1, indicating a low impact of this correlation on the results of the multivariable model.

**Fig. 1:**
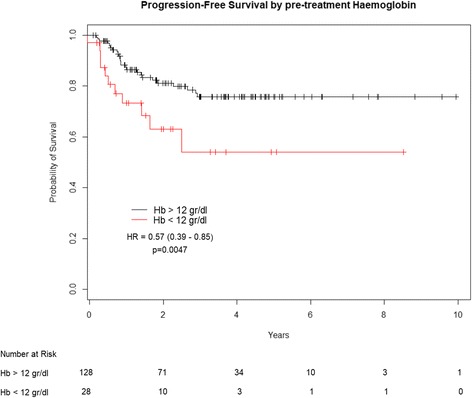
Progression free survival by baseline hemoglobin level

**Fig. 2:**
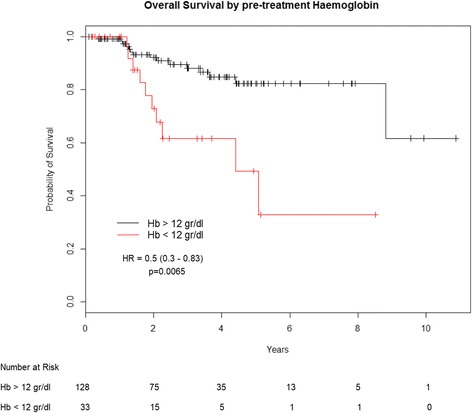
Overall survival by baseline hemoglobin level

**Fig. 3:**
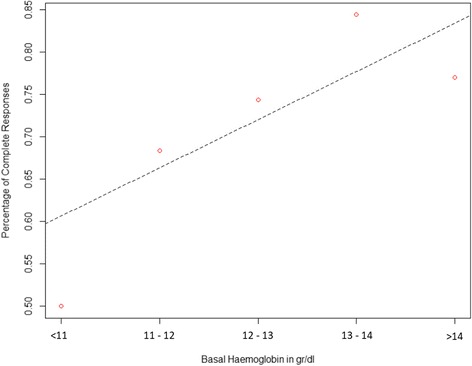
Complete response rate plotted against baseline hemoglobin level

Employing a cut-off point at Hb =12 g/dl, patients with pre-treatment Hb ≥ 12 g/dl had a 5-year PFS of 82.2%, compared to 29.3% for those with Hb < 12 g/dl (HR:0.57; 95% CI: 0.39–0.85; *p* = 0.0047) (Fig. 1). Five-year OS was 82.2% for patients having Hb ≥ 12 g/dl and 32.8% for those having baseline Hb < 12 g/dl (HR:0.50; 95% CI: 0.30–0.83; *p* = 0.0065) (Fig. 2). Weighted linear regression between CR rate and mean Hb values, showed a positive trend, with the likelihood of response increasing at higher Hb values (*p* = 0.11). We categorized patients in our cohort in 5 groups according to baseline Hb levels (< 11 g/dl; 11–12 g/dl; 12–13 g/dl; 13–14 g/dl; > 14 g/dl). Analyzing the slope of the linear regression curve and related intercept, the lowest Hb level category had a 55% chance to achieve a CR after CT-RT. Interestingly, this likelihood increased by 5.6% for every single-unit (g/dl) increase in Hb level (Fig. 3).

**Fig. 4:**
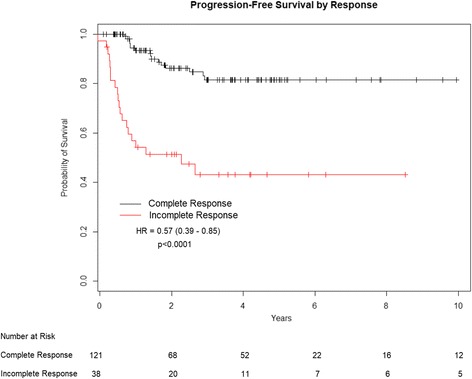
Progression free survival by response to concurrent chemo-radiation

**Fig. 5:**
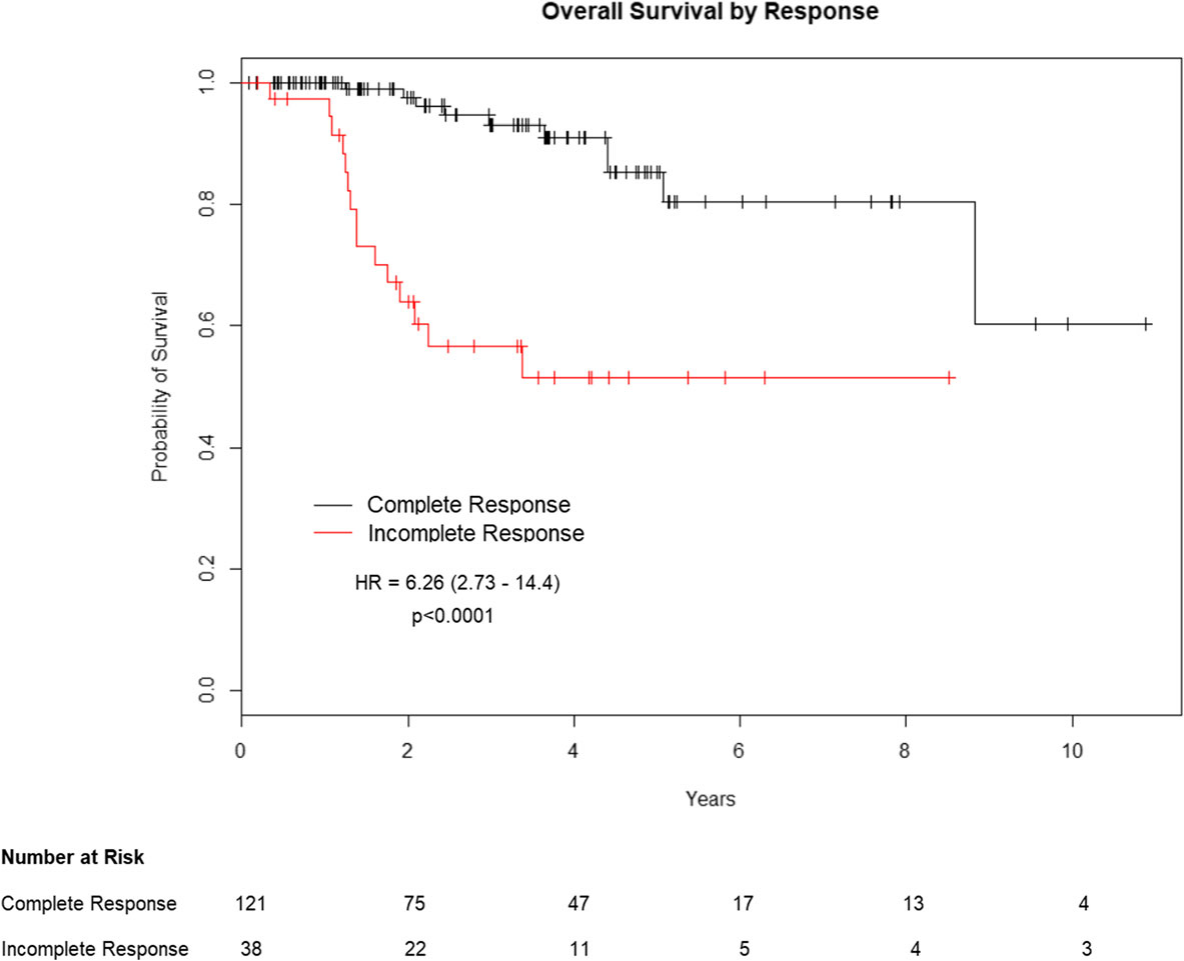
Overall survival by response to concurrent chemo-radiation

Comparing outcomes according to response to treatment, both the 3- and 5-year PFS were 81.5% for patients achieving a CR compared to 43% for patients with incomplete response (Fig. 4). Three- and 5-year OS rates for the same response stratification were 93.1% and 85.4%, 56.6% and 51.4%, respectively (Fig. 5).

## Discussion

As pointed out by Glynne Jones et al., prognostic factors have to be intended as specific measurable characteristics that can be easily obtained and quantified during observation within a certain population to be potentially correlated to measures of clinical outcomes [11]. In anal cancer, prognostic factors have been described within retrospective series or randomized prospective phase III trials [9, 11, 12]. Some depend on tumor characteristics such as primary tumor dimension and nodal involvement, while others are intrinsic to the patient as for example gender. Hemoglobin level is a patient-related clinical factor that has not been extensively explored in anal cancer patients [15]. The correlation between Hb concentration and tumor oxygenation is well-established in several tumor types, with head and neck cancer being a paradigmatic example. [21, 22]. Anemia may enhance tumor hypoxia, increasing tumor cell radio-resistance and leading to a potentially more aggressive tumor phenotype [11]. This is well-known, for example, in cervical cancer, but evidence is also present for anal malignancies [23, 24]. Interestingly, in the RTOG 98–11 phase III trial, which explored the role of cisplatin concurrent to radiation and that of maintenance therapy, patients having levels of Hb below 10 g/dl before randomization were excluded upfront from the study, because of the potential dismal prognosis [7]. In this sense, international clinical guidelines (such as those of the National Comprehensive Cancer Network) suggest the use of blood transfusion in symptomatic patients with Hb levels below 10 g/dl, in order also to potentially enhance tumor re-oxygenation [25]. Our data seems to confirm these findings. On univariate analysis, baseline Hb levels, considered as a continuous variable, had a significant correlation to both PFS (HR:0.57;95%CI:0.39–0.85;*p* = 0.049) and OS (HR:0.53;95% CI:0.29–0.96; *p* = 0.047). Conversely, on multivariate analysis, baseline Hb was significantly correlated only to OS (HR:0.50;95% CI:0.31–0.83;*p* = 0.0051), but not to PFS (HR:0.80;95% CI:0.48–1.34;*p* = 0.40). The discrepancy observed for the correlation between pre-treatment Hb and OS vs PFS prompts to interrogate whether baseline Hb is a real independent prognostic factor or if it is just a surrogate parameter for patient’s comorbid conditions or worse prognostic outcome. Nevertheless, it should be noted that the results seems to suggest the presence of a correlation trend between Hb and PFS and hence, our hypothesis is that this observation can be due to the slenderness of the sample size in our study. We also cannot rule out an effect related to competing causes of death, as low Hb levels could be related to a ‘frail’ patient phenotype or worse clinical conditions predisposing to a higher likelihood of death from any cause, not strictly related to cancer. We also have to notice that we could detect a significant correlation between nodal involvement and baseline Hb levels. Again, we cannot fully rule out the possibility that this correlation could explain, at least partially, the poor PFS and OS related to Hb levels. However, both nodal statuts and Hb levels were comprised within the multivariable model, showing independent statistical significance. Moreover, the VIF for both PFS and OS showed very low values (< 1.1), strongly suggesting a limited influence of this correlation on the final outcome results. In our analysis, we employed a cut-off point at Hb =12 g/dl, which was able to allocate patients to different prognostic classes for both 5-year PFS (82.2% for Hb ≥ 12 g/dl vs 29.3% for Hb < 12 g/dl) and OS (82.2% for Hb ≥ 12 g/dl vs 32.8.% for Hb < 12 g/dl). Our data are in line with those of Roldan et al. who found a significant correlation between pre-treatment Hb levels and PFS and OS on univariate analysis, in a series of 72 anal cancer patients treated with concurrent CT-RT [15]. On multivariate analysis, lowest quartile pre-treatment Hb was a predictor for PFS, while pre-treatment Hb level was a significant predictor for OS. Interestingly, response to treatment at 3 months (together with nodal involvement and performance status) was confirmed as a significant predictor for both PFS and OS. Using baseline Hb values below 12 g/dl as a cut- off point (only 10% of patients were below the cut-off), Hb levels remained significantly associated to OS (log-rank *p* = 0.003) and PFS (log-rank *p* < 0.0001). In general, patients with pre-treatment Hb values in the lowest quartile had significantly worse PFS and OS than those whose values were in the 3 higher quartiles. A similar threshold was also found by Kapacee et al., in their series of 148 anal cancer patients treated CT-RT within the ACT-II trial (50.4 Gy/28 fractions delivered over 38 days concomitant to 5-FU and either MMC or DDP), where a pre-treatment Hb level < 13 g/dl was found to predict for lower distant metastasis and cancer-specific survival (*p* < 0.05) [26]. Given the impact of both CR and baseline Hb level on treatment outcomes, we tried to quantify the relationship between these 2 clinical variables. After clustering patients in different groups based on baseline Hb levels, we analyzed the slope of the linear regression curve and the intercept. The lowest Hb level category has a 55% chance to achieve a CR after CT-RT. Interestingly, this likelihood increases by 5.6% for every single-unit (g/dl) increase in Hb level. In our series response to treatment was found to be an independent predictor of PFS and OS, while baseline Hb level was found to independently predict OS but not PFS. Nevertheless, clinical response and baseline Hb seem to have a synergistic effect in determining survival, with higher pre-treatment Hb increasing the chance to achieve a CR and thus potentially affecting survival. This is in line with data coming from the ACT-I randomized phase III trial, which compared exclusive radiation to 5-FU/MMC-based concurrent CT-RT. In the analysis of prognostic factors performed within the study, baseline Hb level was shown as an independent prognostic factor for anal cancer-related death [11]. After adjusting for sex and lymphnode status, Glynne-Jones et al. demonstrated that, on average, a single-unit (g/dl) increase in Hb was associated to a 19% reduction in the risk of anal cancer death [11].

In our study pre-treatment Hb levels were significantly correlated to overall survival. The HR we found at multivariate analysis (HR:0.5), was similar to that previously reported in other oncological settings [14]. That means that every patient has a 50% decrease in the risk of death at each time-point for a single unit increase in Hb. Interestingly, such an effect is also observed for Hb levels above 11 g/dl, suggesting that even a slight, subclinical decrease in baseline Hb could significantly impair prognosis. Hemoglobin level is also associated to the likelihood of achieving a CR, which was found to be as the strongest independent prognostic factor for survival. The chance to achieve a CR increases by 5.6% for every single-unit (g/dl) increase in Hb level. On the contrary, lower Hb levels do predict for worse outcome. We cannot fully exclude other causes for the dismal prognosis seen in anemic patients. Baseline hemoglobin levels can be a mere consequence of a more aggressive biology of the tumor or a surrogate for patient’s frailty or comorbid state. But at the same time baseline Hb levels seem to strongly predict for a higher likelihood to achieve a complete response to treatment. Probably, both aspects play a role in the final clinical outcome of the patient.

## Conclusions

Our study has several limitations. At first, the small sample size did not allow us to perform subset analyses to better investigate the correlation between baseline Hb levels and other clinical variables potentially affecting treatment results. Secondly, the retrospective frame did not allow us to robustly check for eventual confounding factors. Another element, potentially influencing the results, is the different RT approach employed in the participating centers (simultaneous integrated boost vs sequential strategies), which implies different overall treatment times. The differential treatment length may be a factor influencing the relative strength in the correlation between baseline Hb and clinical outcomes. Moreover, we were not able to discriminate neither the different causes of anemia in our patients (iron deficiency, bleeding, nutritional status or chronic disease) nor the consequent treatment strategies employed to correct the deficit. This would have helped to better put into perspective the correlation between anemia and clinical outcomes [27]. Finally, we were not able to consistently track treatment breaks and modifications to have an idea on the eventual association with Hb levels and to monitor the clinical meaningfulness of the parameter [28, 29]. Nevertheless, we were able to demonstrate that low baseline Hb levels are correlated to a higher likelihood to experience an incomplete response after treatment. Hence, objective response after CT-RT should be carefully monitored in these patients. Considering the poor prognosis associated to a lack of objective response to treatment and the significant impact on quality of life due to colostomy for those submitted to salvage surgery, we encourage oncologists to consider as a clinical priority the preservation of adequate Hb levels before starting CT-RT, but, possibly, during all phases of active therapy. To fully confirm this preliminary hypothesis, a prospective study having anemia correction as intervention is needed.

## Abbreviations

5-FU: 5-fluorouracil
CR: Complete response
CT: Chemotherapy
CT-RT: Chemo-radiation
DDP: Cisplatin
DFS: Disease-free survival
Hb: Hemoglobin
HR: Hazard ratio
LRR: Locoregional recurrence
MMC: Mytomicin C
MRI: Magnetic resonance imaging
OS: Overall survival.
PD: Progressive disease
PET: Positron-emission tomography
PFS: Progression-free survival
PR: Partial response
RT: Radiotherapy
SD: Stable disease
SIB: Simultaneous integrated boost
SLNB: Sentinel lymphnode biopsy
